# Investigating increasingly complex macromolecular systems with small-angle X-ray scattering

**DOI:** 10.1107/S2052252514020843

**Published:** 2014-10-21

**Authors:** Bente Vestergaard, Zehra Sayers

**Affiliations:** a Department of Drug Design and Pharmacology, University of Copenhagen, Universitetsparken 2, Copenhagen, DK-2100, Denmark; b Faculty of Engineering and Natural Science, Sabanci University, Orhanli, Istanbul Tuzla 34956, Turkey

**Keywords:** biological solution small-angle X-ray scattering (BioSAXS), synchrotron radiation, beamlines, structural complexity, biostructural research

## Abstract

The biological solution small-angle X-ray scattering (BioSAXS) field has undergone tremendous development over recent decades. This means that increasingly complex biological questions can be addressed by the method. An intricate synergy between advances in hardware and software development, data collection and evaluation strategies and implementations that readily allow integration with complementary techniques result in significant results and a rapidly growing user community with ever increasing ambitions. Here, a review of these developments, by including a selection of novel BioSAXS method­ologies and recent results, is given.

## Abstract 2

A review of recent and ongoing development and results within the field of biological solution small-angle X-ray scattering (BioSAXS), with a focus on the increasing complexity of biological samples, data collection and data evaluation strategies.

## 1. Introduction   

Hardware and software advances at large-scale facilities are continuous science drivers, enabling substantial advances in life science research. Over the last few decades, such development has revolutionized the use of biological solution small-angle X-ray scattering (BioSAXS), resulting in a significant increase in publications from this research field (Graewert & Svergun, 2013 ▶). BioSAXS is a versatile and powerful method, which allows direct derivation of structural parameters from biomacromolecular samples in solution, notably with very few restrictions on experimental conditions (Svergun *et al.*, 2013 ▶). Compared with high-resolution structural biology methods such as macromolecular crystallography (MX) and nuclear magnetic resonance spectroscopy, BioSAXS is a low-resolution method. However, by advanced software implementation and utilizing additional information on, for example, available high-resolution information from individual domains, data can be evaluated in a discriminative fashion, hence the resolution limit of the method becomes debatable as discussed, for example, by Rambo & Tainer (2013 ▶). This discriminative power has been used for decades to validate or elaborate on controversial high-resolution structures [see *e.g.* Bilgin *et al.* (1998 ▶) and McCord *et al.* (2013 ▶)]. Of even greater importance is the complementarity compared with most other biostructural methods as samples used in BioSAXS are analysed in solution, allowing for the analysis of mixtures, flexible systems and developing processes, potentially in a time-resolved manner and in response to changes in the experimental conditions. Several excellent recent reviews provide an overview of BioSAXS analysis (*e.g.* Tuukkanen & Svergun, 2014 ▶; Pérez & Nishino, 2012 ▶). Here, we reflect on the current development in the field, emphasizing a few recent spectacular results, while providing a scope for the future potential of BioSAXS.

## 2. Automation is a keyword   

**Figure 1:**
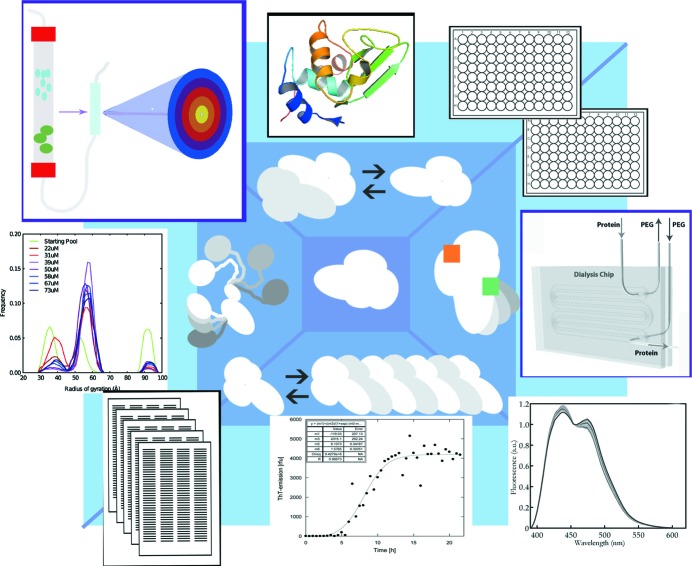
BioSAXS analysis of complex samples. Purple center: data collection from monodisperse samples and routine *ab initio* shape reconstruction. Blue panel: some states of complex mixtures. Light-blue periphery: examples of novel approaches. Mixtures of oligomeric states or less-than-pure samples may be analysed using size-exclusion chromatography (top left) and available high-resolution data may be utilized in modeling (high-resolution structure of equine lysozyme (2eql) is shown as an example). High-throughput data collection for samples that are sensitive to experimental conditions is facilitated using automated sample loading, *e.g.* from 96-well plates (top right), or by using microfluidic sample environments (middle, right). Microfluidic dialysis (Skou, Skou *et al.*, 2014 ▶) can be used for titrating small-molecule ligands (green square: ligand binding; orange square: allosteric modulator) and possible structural changes can be monitored (bottom right). Complex processes such as large-scale polymerization or protein fibrillation (bottom) can be followed, and complementary biophysical data (*e.g.* spectroscopy data) can be used in analyses. Meta data (bottom, left) from previous measurements can be used to optimize data collection strategy or to support data evaluation. Highly flexible protein structures (left) can be analysed by applying ensemble modeling [example from Møller *et al.* (2013 ▶)].

The last few years have seen significant developments, fuelled by excellent BioSAXS beamline research groups and collaborative efforts, in the level of automation during BioSAXS data collection (Fig. 1 ▶). At advanced beamlines, advanced fluid handling robots efficiently load sample volumes of approximately 10 µl (Round *et al.*, 2008 ▶; Martel *et al.*, 2012 ▶; Classen *et al.*, 2013 ▶; Pernot *et al.*, 2013 ▶) and ensure thorough cleaning of the sample cell, high reproducibility, minimal human error and high sample throughput. In combination with significant advances in automated data reduction, processing and analysis, this provides the user with an on-the-fly overview of data quality, the overall biophysical and structural parameters, and in some cases even initial *ab initio* models (Franke *et al.*, 2012 ▶).

The concurrent progress in molecular biology methods means that research groups now provide samples that were previously difficult, if not impossible, to produce in adequate quantity and quality for systematic structural analyses. The availability of high-throughput low-volume data collection is crucial for such projects. Importantly, these data collection strategies also immediately provide the opportunity to increase the experimental complexity. Rather than analysis of a given macromolecular system in one selected experimental condition, it is becoming increasingly possible to screen structural effects of variations in experimental conditions. Ultimately, given the right software development, it should become possible to systematically search for optimized experimental conditions, providing the highest quality of structural data for a given number of structural states, and thus to screen the potential structural space of a biological system (Toft *et al.*, 2008 ▶). Supported by such approaches, the bio­structural community is experiencing a change in the way we think; moving from considering macromolecules as serial changes between static structures, towards an understanding and investigation of macromolecules as a statistical distribution of indefinite numbers of structural states, each represented by a certain probability, and being highly sensitive and responsive to the environment.

MX software developers spearheaded the implementation of a web-based laboratory information management system for synchrotron data, named Information System for Protein Crystallography Beamlines (ISPyB; Delageniere *et al.*, 2011 ▶). As is the case for an increasing number of SAXS users, MX users collect thousands of data sets related to the same research project. Therefore, when collecting new data, access to previously collected data becomes crucial in order to optimize the current experiment, and to benefit from the available metadata. This includes sample tracking, comparison of experimental variations, data quality evaluation, data archiving and comparative data processing. Integration of BioSAXS data to this system, which would facilitate interpretation of results from rapidly increasing number of experiments performed on synchrotrons today, is being tested at the ESRF (Round *et al.*, 2014) and it is also being installed at the EMBL-Hamburg, Diamond and SOLEIL facilities (Fig. 1 ▶).

## 3. Microfluidics: optimization of complementary data collection   

Improved fluid handling efforts also extend to the development of microfluidic sample environments for BioSAXS data collection. On top of enabling even further reduced sample volumes and the general benefits of automation outlined above, the modular nature of microfluidic devices offers an opportunity to integrate complementary analysis modules on the chip. This may include *e.g.* concentration estimates from ultraviolet–visible spectroscopy (UV–vis) (Lafleur *et al.*, 2011 ▶) and Raman spectroscopy (Haas *et al.*, 2014 ▶), thereby resulting in simultaneous collection of biophysical information and structural data. In the above cases, simultaneous monitoring of the SAXS profiles, protein concentration and chemical fingerprint of the samples facilitates monitoring of, for example, radiation damage, protein folding state and average oligomerization state.

Several successful SAXS microfluidic sample environments are reported in the literature (Pollack *et al.*, 1999 ▶). Using a similar setup Brennich *et al.* (2011 ▶) were able to capture structural data from the early assembly states of intermediate filaments, which would not have been accessible using a conventional sample setup. Likewise, Møller *et al.* (2013 ▶) characterized a complicated mixture of different oligomeric states of a partially flexible protein involved in mitochondrial metabolism, by decomposing data from a number of experimental conditions obtained by microfluidic titration. One recent development reports the merging of sample dialysis with SAXS data collection (Skou, Skou *et al.*, 2014 ▶), thereby eliminating the risk of overly concentrating the macromolecular samples prior to data collection, and allowing very gradual changes of experimental conditions during data recording (Fig. 1 ▶). It seems likely that future sample environments will add sample modifying modules, *e.g.* micropurification devices, temperature variation, pressure cells or other more or less exotic devices, immediately prior to SAXS data collection. Both existing and such future devices allow the triggering of structural changes. Current state-of-the-art time-resolved microfluidic solution scattering experiments utilize spatially defined time resolution with ultrafast mixers originally advanced by Pollack *et al.* (1999 ▶). In the future, even higher time resolution may be achieved, as demonstrated in non­microfluidic environments by pump–probe approaches using either small- (Cho *et al.*, 2013 ▶) or wide-angle scattering (Cammarata *et al.*, 2008 ▶) which may be coupled with microfluidics devices. Although the topic is beyond the scope of this review, it should at least be mentioned that with the anticipated future advances in X-ray free-electron-laser experiments possibilities for studies of complex samples displaying ultrafast structural dynamics will increase dramatically.

One significant area in recent efforts to advance data collection strategies is the cryoprotection of samples during data collection. This development is not trivial, for several reasons: firstly, addition of cryoprotectants increases the scattering background arising from the solvent, and hence decreases the contrast between protein and solvent, resulting in poorer signal-to-noise ratios in the data. Secondly, the addition of any chemical/excipient to a macromolecular solution has the potential to modify the structural parameters of the macromolecule of interest. The latter can be tested by comparing cryodata with low-dose data from nonprotected samples, which however increases the complexity of both data collection and analysis. Current efforts rely on nanolitre sample films dispersed in windowless sample containers, which are cooled while including cryoprotectants in the sample buffers (Meisburger *et al.*, 2013 ▶), such as those that have been developed over decades for MX. This approach thus allows for even further reduction in sample consumption, with the price of including a small pathway of air around the sample, which at present is not vacuum compatible. Future developments in cryo-SAXS are very important, for allowing data collection from highly radiation-sensitive species, as well as for further advancement of mail-in high-throughput remote data collection protocols (Meisburger *et al.*, 2013 ▶).

## 4. Dynamic structures and mixtures   

An important quality of BioSAXS data is the option to analyse mixtures. Evidently, such samples must be well characterized, as mixtures increase the complexity of the sample, and thus the number of structural parameters to investigate. SAXS data are often said to be underdetermined and no unambiguous models can be derived from SAXS data (Koch *et al.*, 2003 ▶). However, SAXS data are derived from all species present in the sample, weighted by their relative volume fractions, which can be used to the advantage of the investigator. Careful sample preparation, for example obtaining data curves from titration experiments, and utilizing prior biochemical information enable successful analysis of highly complex samples, and characterization of individual species. The full BioSAXS-based recording of scattering from all individual molecules in solution means that careful sample preparation, for example, by including a number of data curves from titration measurements and/or by including prior knowledge, enables successful analysis of highly complex samples, and potential characterization of individual species. Increasingly complex software is continuously developed, and can be applied by expert users [one recent overview is provided by Graewert & Svergun (2013 ▶)]. Several such interesting investigations are seen in the recent literature, and only a small subset of examples is included below.

### 4.1. In-line protein purification   

An important development in BioSAXS is the availability of in-line purification setups at several advanced synchrotron beamlines (Fig. 1 ▶). Originally developed within an international synchrotron network (David & Pérez, 2009 ▶), in-line size-exclusion chromatography (SEC), in particular, now enables the analysis of unstable species which are difficult to preserve until the time of data collection. X-ray beam quality and fast detectors, coupled with automated analysis, provide ideal conditions for fine-slicing of the data, meaning that measurements can be obtained from the individually purified species. A common use is for separation of smaller fractions of aggregated protein from the species of interest, thereby significantly improving data quality, although the inherent dilution of the sample is a downside of the method. A few important examples of more advanced applications of the method include the study of actin self-assembly (Didry *et al.*, 2012 ▶) and the first example of the analysis of a SEC-purified micelle-embedded membrane protein (aquaporin-0), elegantly facilitated by the optimized background subtraction using data from the micelle–detergent containing buffer eluting immediately prior to the membrane protein fractions (Berthaud *et al.*, 2012 ▶). Alternative purification strategies coupled with SAXS data collection have to our knowledge not yet been published, but they are anticipated to appear. Standard purification procedures, such as ion-exchange chromatography, may separate different functional states of a given protein and have the additional advantage that the eluting sample is at high concentration. Also in the case of ion-exchange chromatography, the buffer eluting immediately prior to the sample must be used for background subtraction.

### 4.2. Membrane proteins and protein–nucleic acid complexes   

Although the above-mentioned study of aquaporin solved the problem of background subtraction, the composite nature of the sample remained an analytical challenge. Membrane proteins must be embedded in either lipid-based vehicles or detergents to stay soluble. As proteins, lipids and detergents have differing electron densities, X-ray-based solution analysis is severely compromised, *e.g.* standard *ab initio* modeling is excluded. In the above study, a geometrical description of the lipids and an independent molecular dynamics modeling of the lipid dynamics were included (Berthaud *et al.*, 2012 ▶). The problem with multiple phases also extends to complexes of proteins and nucleic acids. Software is available for multiphase bead modeling [the first program being *MONSA* (Svergun, 1999 ▶)], originally intended for contrast variation in neutron data, but also recently applied to such a nucleic acid–protein complex based on X-ray data from various subcomplexes (Mallam *et al.*, 2011 ▶). Other multiphase modeling programs are available [such as *Igor* (Kline, 2006 ▶) and *WillItFit* (Pedersen *et al.*, 2013 ▶)] reflecting a general effort for software development in several leading international groups.

As an interesting development, recently significant efforts have been put into developing nanodiscs (Denisov *et al.*, 2004 ▶) as versatile tools for X-ray- and neutron-based solution studies of membrane proteins (Maric *et al.*, 2014 ▶). Both software and samples have been developed, incorporating partially deuterated lipids and protein species, ultimately resulting in nanodiscs that when applying suitable deuteration levels of the solvent become nearly invisible in the scattering data (playfully named as *stealth carriers* of the protein species). This means that, in principle, the incorporated protein can be treated like any soluble protein (Maric *et al.*, 2014 ▶). Although analysis of X-ray solution data from such composite material remains challenging, such samples are equally difficult to study by other conventional structural methods, and the current methodological development is of great importance for future advances in these research areas.

### 4.3. Equilibria and mixtures of structural states   

Not all mixtures of macromolecular states can be analysed by purification. Mixtures of different structural species exist in equilibrium in the solution, hence separation of individual species will to some extent influence the distribution. In the above-mentioned cases, the time window of analysis suffices to successfully separate nonspecific aggregates from individual representative species. However, in several cases this is not possible. Examples include allosteric transitions of protein structures such as the study of aspartate transcarbomoylase, where the allosteric-induced transition between structural states includes an intermediate state revealed by SAXS analysis (Guo *et al.*, 2012 ▶) or protein fibrillation processes, where the delicate balance between a number of structural states is highly sensitive to experimental conditions. In both of the above cases, the solutions should be studied without disturbing the equilibria. Only a few examples of fibrillation analyses have been published (Vestergaard *et al.*, 2007 ▶; Oliveira *et al.*, 2009 ▶; Giehm *et al.*, 2011 ▶; Vetri *et al.*, 2013 ▶), offering unique insight into the structural details of important intermediately formed structures. Other examples of similar complexity include frataxin analysis (Söderberg *et al.*, 2011 ▶; Söderberg *et al.*, 2013 ▶) or the aforementioned study of intermediate filament assembly (Brennich *et al.*, 2011 ▶). Such analysis puts rather extreme demands on sample quality and reproducibility, and is not likely to become standard in the near future. It is, however, important to emphasize how BioSAXS analysis uniquely offers the opportunity to study such highly challenging structural systems, and given the importance of addressing the structural aspects of these, it is likely that we will see a continuous effort within analysis of such complex processes. Ongoing efforts include both software (Petoukhov *et al.*, 2012 ▶) and the aforementioned inclusion of complementary concurrently measured biophysical data (Haas *et al.*, 2014 ▶) to objectively support the data analysis (Fig. 1 ▶).

### 4.4. Flexible structures and intrinsically disordered proteins   

More recently, it has been acknowledged that eukaryotes in particular express a large number of intrinsically disordered proteins (IDP) (Uversky, 2014 ▶) implicated in a variety of cellular processes, typically involving transient protein–protein interactions and functional regulation (Wright & Dyson, 2009 ▶; Babu *et al.*, 2011 ▶). Such proteins thus are not in one particular well defined conformation, but rather adapt to a wide distribution of structures. This means that this class of proteins is impossible to analyze using classical structural biology methods: protein crystallization requires monodisperse protein solutions and a crystal structure describes one (or very few) conformation(s) captured in the crystal lattice. Likewise, although NMR can provide useful information about the local structure of IDPs, classical analysis did not yield information about the global structural dynamics. However, such structural ensembles may be analysed from SAXS data. Originally pioneered by Bernadó *et al.* (2007 ▶), and often in combination with complementary NMR data (Jeffries *et al.*, 2011 ▶), IDPs are analysed by applying different variants of ensemble modeling approaches (Pelikan *et al.*, 2009 ▶; Bernadó & Svergun 2012 ▶; Berlin *et al.*, 2013 ▶; Varadi *et al.*, 2014 ▶). Fitting to SAXS data and applying different optimization principles [a genetic algorithm is used in the original approach by Bernadó *et al.* (2007 ▶)] an optimized subset of structures (an ensemble) is selected from very large pools of potential structures. The average scattering from the ensemble represents the collected structural features of the distribution of structures in solution. This provides intriguing insight into the principles behind functionality embedded within structural flexibility and the sensitivity to experimental changes (Różycki *et al.*, 2011 ▶). Another example is the analysis of the Tau protein, whose structural conversion is involved in the progress of neuro­degenerative diseases. Here, ensemble analysis revealed both the presence of short-range (residual domains) and long-range contacts adapted to different degrees in the collected ensemble of structures (Mylonas *et al.*, 2008 ▶).

Other proteins are not fully IDPs, yet contain significant unfolded stretches, either in the peptide chain termini (see Wells *et al.*, 2008 ▶) or in between protein domains (Fig. 1 ▶). In the latter case, this evidently causes significant flexibility influencing, for example, the formation of protein–protein contacts. Even proteins, which are normally considered to be structurally stable and hence typically characterized by MX, are highly dynamic entities exhibiting varying degrees of local or global structural changes. Solution scattering offers an important opportunity to capture and characterize such inherent protein structural dynamics, if including also wide-angle scattering, hence monitoring the structural parameters describing the dynamics of short-range distances in macromolecular structure. Examples include the monitoring of structural stability (Fischetti *et al.*, 2003 ▶) and the characterization of protein–ligand interactions (Minh & Makowski, 2013 ▶). The method is anticipated to develop significantly in the nearest future, also potentially including triggered structural changes and time-resolved analysis, as exemplified by the analysis of hemoglobin structural changes (Cammarata *et al.*, 2008 ▶).

## 5. Including prior knowledge and hybrid analysis   

If attempting high-resolution characterization of macromolecules in solution, a particularly important but not yet fully resolved aspect must be taken into account: solubilized macromolecules interact specifically with solvent molecules, resulting in a solvent layer (primarily of water molecules), surrounding the particle. Since such an organized solvent layer will have a different electron density compared with the bulk solvent, it will contribute significantly to the particle scattering. This topic has been addressed for decades (Svergun *et al.*, 1998 ▶) and substantial progress was achieved when Svergun and coworkers developed software for efficient calculation of theoretical solution scattering patterns from high-resolution structural data, including also a solvent layer of variable width and average density (Svergun *et al.*, 1995 ▶). Several groups have implemented variations of the calculation of scattering patterns from atomic structures, and the water layer representation (Grishaev *et al.*, 2010 ▶; Poitevin *et al.*, 2011 ▶; Putnam *et al.*, 2013 ▶) and these efforts represent important and challenging development which is prerequisite for potential future high-resolution modeling from solution data. The analysis of protein water layers also originally included neutron scattering data (Svergun *et al.*, 1998 ▶) exemplifying the need for complementary data in challenging cases. The inclusion of high-resolution models from MX and NMR indeed revolutionized the BioSAXS (and to a lesser extent the BioSANS) field.

An interesting and developing application of SAXS is found in anomalous (solution) small-angle X-ray scattering (ASAXS) which is a tool for probing the dynamic features of a structure by determining positions of specific metal groups or atoms within the macromolecule in solution (Makowski *et al.*, 2012 ▶). A further example for hybrid approaches is found in the example of a combination of ASAXS with all-atom molecular dynamics simulations to map metal ions that are part of the structure of short RNA molecules. In a recent study, this approach was applied to accurately predict counterion properties and fluctuations around the native structure. The model thus obtained for the short RNA pseudoknot by the two techniques deviated from crystal structure predictions. This result highlights the importance of analyzing structures under conditions free of constraints introduced by crystal contacts (Kirmizialtin *et al.*, 2012 ▶).

Today, hybrid methods including biophysical and structural data from various sources are applied to the analysis of particularly challenging cases, an approach which has the clear advantage of reducing the ambiguity in the analysis of solution scattering data. One recent example is seen in the elegant analysis of leptin receptor complexes (Moharana *et al.*, 2014 ▶), where data are included from high-resolution structural analysis, SAXS and microscopy analysis. Another example combines NMR, homology modeling and SAXS data, developing a quasi-atomic resolution model of the multicomponent polyketide synthase enzymatic complex (Davison *et al.*, 2014 ▶). The Sali group has advanced software, implementing hybrid data analysis (Russel *et al.*, 2012 ▶), resulting in detailed analysis of the yeast RNAPII system. Future ISPyB efforts could indeed include the full implementation of structural and experimental information from all complementary methods available, hence partially automating the incorporation of data from several sources.

## 6. Time-resolved SAXS   

With the advance of new pixel detectors, not only has the data quality from solution scattering significantly improved (Kraft *et al.*, 2009 ▶; Johnson *et al.*, 2012 ▶), but also, the option to perform time-resolved data collection with timeframes that are directly relevant for individual protein structural changes has taken a quantum leap forward. Data quality will improve yet further with the next generation of detectors that will soon reach the beamlines. In this context, demands for sophisticated sample handling conditions increase dramatically, if the field aims to take full advantage of these new technological possibilities. If experimental triggering of a structural conversion is not instant throughout the sample, the resolution, both spatially and in time, will be compromised. Current examples use pump–probe approaches triggering a photosensitive sensor with powerful lasers (Cammarata *et al.*, 2008 ▶; Cho *et al.*, 2013 ▶). Clearly, in order to investigate proteins and other macromolecules that lack such an intrinsic photosensitive probe, further development in the field is necessary. Again, microfluidics may provide a solution by reducing transfer times of triggering factors, simply by diminishing the diffusion distances through the sample volume illuminated by the X-ray beam. Hence, further development of improved time-resolved SAXS is a good example of how technological developments on synchrotrons drive science opportunities: the future expected availability of increasingly collimated and intense (sub-)micro-sized X-ray beams, ultrafast detectors and advanced microfluidic sample platforms, is anticipated to facilitate the structural analysis of increasingly complex samples.

## 7. A word of caution   

Given the availability of highly complex samples, and the urge to implement increasingly complex analysis of structural solution data from these systems, a word of caution is necessary. SAXS data are quite easily mis- or over-interpreted, and cross-validation of results with data from complementary methods is often necessary to eliminate the danger of unjustified conclusions. Current publications exemplify the need for guidelines, *e.g.* including recent reviews on ‘how-to-do’ at synchrotron beamlines (Dyer *et al.*, 2014 ▶; Skou, Gillilan *et al.*, 2014 ▶), or the widely used SAXS publication guidelines (Jacques *et al.*, 2012 ▶). Great effort has gone into developing common formats and guidelines for the deposition of SAXS data and derived models (Trewhella *et al.*, 2013 ▶), much like the existing Protein Data Bank making MX data, structural models and experimental details publicly available.

While the BioSAXS community has virtually exploded in numbers over the recent years, the importance of expert training, currently taking place mostly *via* international courses facilitated by (primarily) the staff at large-scale infrastructures, cannot be overstated. The advanced BioSAXS synchrotron beamlines remain the most powerful centers of software and hardware development, where the current close interaction between users and beamline staff ensures a highly synergetic scientific environment, which must be protected and nourished into the future. It is important that automation and high-throughput setups do not in turn create a situation where joint forces *via* expert sample preparation and expert analysis are no longer prerequisite for analysis of challenging data. Future developers of mail-in options for BioSAXS data collection, for example, or highly automated analysis procedures could consider how to maintain a close relationship between beamline staff and life science users, in order not to lose the current fruitful bilateral inspiration between the communities. With continued proper caution from the decision makers influencing the field, the great advances that we have seen over the last few decades in BioSAXS, could very well increase even further in magnitude, and deliver future results of great importance to the biostructural field.
